# Report of Twenty-seven Cases of Puerperal Peritonitis

**Published:** 1856-04

**Authors:** Robert K. Smith

**Affiliations:** Chief Resident Physician, Philadelphia Hospital


### MEDICAL EXAMINER.

NEW SERIES.—NO. CXXXVI.—APRIL, 1856.

### ORIGINAL COMMUNICATIONS.

*Report of Twenty-seven cases of Puerperal Peritonitis.* By ROBERT K. SMITH, M. D., Chief Resident Physician, Philadelphia Hospital.

*Philadelphia Hospital*, March 16, 1856.

DR. HOLLINGSWORTH :—

In the annexed report of twenty-seven cases of puerperal peritonitis, you will find a full history of the disease, from its first appearance in the wards of this hospital up to the present date, including a case that I attended in the family of a friend who lives near the institution.

It is not my object, in making you this communication, to present my own views or speculations upon the nature, pathology and treatment of this form of disease; but simply to detail, as accurately as possible, the facts in the different cases as they occurred, in order that the profession may be put in possession of the material, from which its members may deduce such theoretical and practical truths as the character, progress, treatment and termination of the cases would seem to justify.

The first five cases were under the immediate care of Drs. Eastman and Shreve, who had charge of those wards up to the first of January, and who have furnished me the notes of their observations and treatment. The remainder of the cases that occurred in the house were attended by Drs. Braxton and Blackford, as the ward physicians. These gentlemen have given me the report of their cases. But it is proper for me to say that I visited all the patients, either with the assistant physicians or in their absence, and carefully watched the whole course of the disease.

There were, previous to the appearance of puerperal fever, and are still a number of cases of erysipelas in the hospital; but there were none nor had been any in the wards where the fever occurred. There were also 17 other obstetrical patients who occupied the same wards, and many of them adjoining beds to the fever patients, that escaped an attack.

After several of the first cases occurred, I ordered the remaining pregnant women to be removed to wards that had not been occupied by obstetrical patients; and some of them were transferred to rooms in the *medical* department of the hospital, immediately upon their admission to the house. This department is in a separate building, more than a hundred feet distant from our obstetrical wards, and where there previously had been no obstetrical cases admitted. These removals, however, had no effect in arresting the progress of the disease; it followed the patients wherever they went, until finally I was induced to discharge from the house every woman who could procure in the city, among their friends or outside of the institution, accommodations during confinement.

There is one other fact worthy of note that I will mention in this connection, and that is, that nearly every child of women having puerperal fever died in convulsions.

Case I. Mary Lacey, a slight, rather delicate looking woman, but in good health; born in Ireland; aged 21; married; delivered Dec. 11th, A. M.; duration of labor 18½ hours; first child; had hour glass contraction and slight adherence of placenta. Saw her at 11 A. M., 13th; had a chill during the previous night, followed by fever and pain in the lower belly; no sleep; restless; lies upon her back; legs extended; complains of pain from pressure upon hypogastrium; skin hot; face flushed; headache and thirst; anorexia; pulse small and quick, 130; not noticeably corded; tongue moist, with a slight coating of white fur.

Sat her up in bed and opened a vein; after drawing about 25

ounces without producing faintness, and the blood failing to run freely, opened a vein in the other arm and drew five additional ounces, when her face blanched and she became faint. Ordered calomel grs. x., to be followed in four hours by ol. ricini f℥j. and poultices constantly applied to the abdomen. At 7 P. M. pain much relieved; can turn upon her side; bowels freely opened; pulse soft, 106; face pale. Ordered calomel grs. iv., pulv. opii grs. ii., every three hours; arrow root for diet.

14th, 11 A. M.—Still pain; has slept; pulse risen to 130, weak; abdomen tympanitic. Ordered 150 leeches to abdomen; continue treatment. After leeching had no further pain, but tympanitis greatly increased; had a deep hectic flush; lay in a half dreamy state, talking occasionally, and when spoken to rousing up, but not able to fix her wandering eyes immediately upon the physician's face.

15th.—Spongy gums, slight ptyalism; discontinued medicine; continued poultices.

16th.—A profuse fœtid diarrhœa came on, with vomiting of frothy, greenish matter, which nothing checked. Died the 17th; no autopsy. Child died the 21st of convulsions.

Case II. Mary McKeown, a raw-boned, slight woman, apparently of good constitution; born in Ireland; aged 22; single; first child; delivered Dec. 11th; labor 12 hours. Saw her on the 13th, immediately after bleeding case No. 1. Had been seized with pain in right iliac region some time that morning; complaining greatly; lies on back, legs extended; flinches upon the slightest pressure over the seat of pain; skin moderately warm; face not flushed, rather an anxious expression; pulse moderately full, soft, 120. Sat her up in bed and bled her about f℥xvi.; she became faint; gave her 10 grs. calomel, to be followed in four hours by an ounce of castor oil; ordered poultices to the abdomen. At 7 P. M. cool, quiet; pulse still frequent. Prescribed calomel grs. vj., opium grs. ii. every three hours.

14th.—Has more fever than yesterday; great tenderness over hypogastrium. Bled her again ℥xvj. and applied 100 leeches to the seat of pain. 7 P. M., covered abdomen with a blister; has not reacted from bleeding; pain continued, sinking rapidly.

15th.—Diarrhœa came on, frequent, copious, black and fœtid, accompanied by vomiting of greenish frothy matter; slight tympanitis appeared twelve hours before death, which occurred on the morning of the 16th, after 12 hours of frightful agony, which no amount of opium would alleviate.

*Autopsy ten hours after death.*—Slight prominence of abdomen, caused by effusion of purulent serum. Masses of pure pus; an ounce or two lying on anterior face of uterus and right ovary; peritoneum slightly, if any, injected, but glazed by a pasty matter that seemed to extend over the entire surface. Intestines somewhat more injected than parietal peritoneum; covered by same exudation. Internal surface of uterus healthy; tissue of uterus firm; color reddish-white; blood coagulated in iliac veins apparently healthy.

Child died of convulsions.

CASE III. Ann Kelly, a large, florid, stout, healthy woman; born in Ireland; aged 23; widow; third pregnancy. Had been transferred from women's venereal wards, where she had been treated for non-indurated chancre. No constitutional treatment had been resorted to. Confined Dec. 13th, M.; suffered from apparent after-pains during the night, which were relieved by an opiate.

14th.—Looks well; pulse quiet; was moved into a damp ward.

15th.—Had light pains in abdomen last night; gone this morning; no pain upon pressure; pulse quiet.

16th, 11, A. M.—Was attacked last night with violent pains in lower belly, extending up the right side to the ribs; skin cool, moist; pulse small, 130; countenance pale and anxious; complains of intense pain of abdomen; cannot bear the slightest pressure. Sat her up in bed and bled her thirty ounces before she became faint; to take immediately 5 grs. calomel and one of morphia. At 7, P. M., pain almost gone; has slept; lies upon her side; skin warm; countenance free from the anxious expression; pulse full and soft, about 100. To take cal. vi. grs., opium iv. grs., every three hours during the night.

17th, 11, A. M.—Has taken 24 grs. opium and 36 of calomel since last night; slept pretty well; condition much as last night; very little pain in the abdomen upon pressure.

18th, 11, A. M.—Slept at intervals; got out of bed and wandered about in search of water; has pain in right side with some difficulty of breathing; percussion dull over lower half of right lung; respiration almost absent, with diminished pectoral resonance; fine crepitation in anterior inferior border of left lung.

19th, 11, A. M. Tympanitis having been gradually coming on, is now excessive. Pulse exceedingly frequent, countenance haggard; blue circles around the eyes; all the symptoms aggravated. She gradually sunk and died on the 21st, after suffering from intense dyspnœa, thoracic and abdominal pain, and incapability of lying down.

The pleurisy was treated with dry cups, blisters, dressed with 6 grs. sulph. morphia, frequently repeated. A similar diarrhœa and vomiting came on previous to death, as occurred in the other cases, which could not be relieved by treatment.

*Post. Mort. Sixteen hours after death.*—Great distension of abdomen. Upon opening the cavity of the peritoneum, there was copious effusion of bloody purulent serum. Parietal and intestinal surface brightly injected and covered with pasty matter. Every portion of the abdomen implicated; external uterine surface for about a line in depth softened; the other portions firm, bluish white; internal surface healthy; uterine sinuses healthy, iliac veins healthy, blood coagulated in them. Intense inflammation of whole right pleura, with about three pints of purulent bloody serum effused; lower half of left pleura inflamed, and about a pint of similar effusion. About one half of lower left lung hepatized; the remainder intensely congested, pouring out freely bloody serum upon being cut.

Child died of convulsions.

Case IV. Bridget Matus, a short, thick set, sanguine woman; of good constitution; born in Ireland; aged 35; married; 1st child; delivered December 17th; duration of labor 18 hours.

19th, 11, A. M. Had a chill last night, followed by fever and intense pain in the lower belly. Skin hot and dry; face flushed; pulse full and strong 125. Has great pain in abdomen upon pressure; can feel the enlarged uterus, which is exquisitely tender to the touch. Sat her up in bed and bled her by a copious jet,—the largest I have yet seen flow from a vein,—at least forty ounces before she became faint. Purged her with calomel and salts; and prescribed calomel and opium. Her pain ceased after the bleeding, and she could in a short time turn over on her side. Abdomen moderately tympanitic; her pulse fell below 100. Treatment continued, and in about five days ptyalism came on without further striking phenomena. Her convalescence was rapid. Child died in its second week of convulsions.

CASE V. Rebecca Montgomery, a healthy woman; born in Ireland; aged 24; married; 1st child; duration of labor seventeen hours. Forceps were applied after the head remained eight hours in the cavity of the pelvis; the patient evincing very considerable prostration; child still-born, weight 10 ℔s., boy; delivered on the 20th.

23d. Was seized with a chill and intense pain in lower portion of abdomen; greatly increased upon pressure. Skin hot and dry; face flushed; pulse 120, small, moderately firm; was bled immediately f. ℥xxx. with relief to local symptoms. Purged with calomel and oil, and calomel and opium given every three hours. Ptyalism came on about the sixth day to a slight degree. Weak, frequent pulse; patient evidently depressed; dry, brown tongue, but no symptoms referable to the abdomen. Gave her quinine and the most nutritious diet. A profuse and extremely fœtid discharge took place from the vagina, and upon examination found an aggravated vaginitis, which seemed confined to its lower portion. The whole pudenda swollen and intensely painful. Under injections of chlorinated soda, this inflammation subsided. On the 30th, she was seized with erysipelas of the face, and transferred to the medical wards, where under a tonic and stimulating treatment she slowly recovered.

The children of Mary Lacey and Bridget Matus, died of convulsions in the ward. Those of Mary McKeown and Ann Kelley, were transferred to the nursery, where they also died.

In none of these women was there a greater suppression of the milk than would have been referable to the treatment. The lochia was not in either case entirely suppressed, nor materially changed in character.

From the 11th, when the first case was delivered, until the delivery of case No. 5, on the 20th, five other women were confined and placed in the same and contiguous wards, but escaped the disease. The five cases of fever, above reported, were placed at first in one ward, three of them on one side, in adjoining beds.

The blood drawn from these patients had moderately soft and large coagula, without the buffy coat, and the serum was greenish yellow.

CASE VI. Mary Lynch, asthenic; born in Ireland; æt. 20; married; 2d child; delivered January 1st.

4th. Had high fever; pulse 120. Hot, dry skin; pain in the iliac region, and slight tympanitis. Bled her ℥xx. and gave 10 grs. calomel, with a grain of opium, followed by oil. Applied hot fomentations to the abdomen, continuously; ordered calomel and opium, each a grain, and ½ grain of p. ipecac. every two hours, which was continued for two days.

6th. Vomiting commenced; pain subsided; had a severe cough, for which syr. ipecac. senega and liq. morph. in mixture were prescribed. Breath and sputa had a gangrenous odor, the treatment was continued with but slight change in the patient until the 8th, when she sank rapidly and died on the 9th.

*Autopsy.*—Found more than a quart of pus in the abdominal cavity, with traces of considerable peritoneal inflammation. The internal walls of the uterus were in a gangrenous state. Child died in convulsions.

CASE VII. Margaret Kelly, small, healthy woman; born in Ireland; æt. 20; delivered on the 28th of Dec.; married; easy labor. Taken on the eighth day of confinement with acute pains in the abdomen; hectic flush; anxious expression; pulse 132; blood drawn to the extent of ℥xxvj.; blood cupped and buffy; hot poultices constantly applied to abdomen; gave 10 grains calomel, followed by oil, and ordered calomel 1 gr., opium ½ gr., and ipecac. ¼ gr., every hour; bleeding relieved the pain; began to sink next morning, and put her on brandy punch. She died in 36 hours after the attack.

*Autopsy.*—Uterus gangrenous; considerable congestion of peritoneum; fully a quart of pus in the peritoneal cavity.

Child died in convulsions a few days after its mother. No unpleasant symptom appeared in this case until the 8th day, and she died on the 10th.

CASE VIII. Mary Ann Hood, a stout, healthy woman; born in Ireland ; æt. 19 ; single ; 1st child ; delivered in eight hours, Jan. 7th. Easy labor, was quite well until the 12th. Taken with chill, headache and fever in the night. The ward physician saw her early the next morning, pulse 150 ; respiration above 40 ; face flushed ; abdomen immensely swollen and very tender to the touch ; dorsal decubitus, knees drawn up. Bled her ℥xii. when she fainted ; had 10 dozen American leeches applied to the abdomen ; gave her 10 grs. of calomel and three of opium, her bowels having been freely opened the night previous. Opium and calomel, each a grain, with a ½ grain of ipecac. were given every two hours, and she slept well. At the end of two days vomiting came on, and a return of the pain in the abdomen ; 8 dozen more leeches were then applied, and the ipecac. withdrawn from the powders. The grain doses of calomel and opium were given every hour, with an occasional dose of 3 grains of opium to relieve her pain. Bled her again to the extent of ℥xxiv. ; bore it well ; continued treatment until the morning of the fourth day with but little change, except an arrest of the vomiting, when she was bled a third time. She lost by venesec. and leeching about 84 ounces of blood. After the third day there was an alternate expression of pain and of pleasure in her face. When not in pain she would laugh and express the opinion that she would soon be well, even until within a few hours of her death ; wine whey was given during the last 24 hours. She died on the 5th day in great agony.

No autopsy ; buried by her friends.

CASE IX. Catharine O'Brien, a stout, healthy woman ; born in Ireland ; æt. 40 ; married ; 3d child ; delivered the 14th at 5 A. M ; labor long and tedious ; forceps case ; child stillborn, weighing 10 ℔s. ; much exhausted after labor ; gave her wine several times ; slept a little until 8 A. M ; so much exhausted that wine was administered frequently throughout the the day. Dozed occasionally during the night.

15th. Abdomen tympanitic ; pulse stronger than yesterday ; above 100 ; not strong enough to justify bleeding. 8 doz. leeches applied to the abdomen. 10 grs. of calomel followed by oil, cal. and opii 1 gr. each every hour. Symptoms improved in the evening ; pulse better ; slept until 11 P. M. ; pains recurred ; pulse 110 ; sat her up in bed and took ℥xvi. of blood ; pulse reduced to 95. No pain; hot poultices ordered to abdomen, and gave an opiate. Very little sleep that night. In the morning easy; abdomen exceedingly swollen and tympanitic; pulse hardly perceptible; evidently dying; gave brandy punch; lived nearly three days in this condition; could neither swallow nor sleep; brandy punch injected into the stomach and rectum; died on the 19th in great agony; friends took the body; no autopsy.

Case X. Mrs. H. A., medium sized, well developed, healthy lady; born in Philadelphia; living near the hospital; æt. 24; 3d child; delivered the morning of the 13th; suffered severely with after pains the first 36 hours; directed for their relief, tr. opii gutt. xl., tr. camph. gutt. lx., three or four times during the day. Diet, cracker panada.

14th. Same condition; no tenderness upon pressure; pulse natural; slept very little last night; looks well; no change in treatment.

15th. Pulse regular; slept none last night; pains violent all night; had hot fomentations applied; lochia suppressed; slight tenderness upon the uterus; prescribed opium grs. xii., camph. grs. xviii., in 12 pills, one every two hours. 9 P. M. Had a chill in the afternoon; violent pain in both iliac regions; pulse 125; skin soft and moist; could not bear the abdomen or iliac regions touched; sat her up in bed and bled her until she fainted; drew about ℥xxx.; pulse fell to 95; prescribed ℞ calomel grs. vj., P. opii. grs. ix., tart. antim. gr. ss., nit. potass ʒss., M. ft. chart. No. vj. One every three hours, to be continued through the night; pain relieved by the bleeding; ordered warm poultices to be kept on the abdomen.

16th, 7, A. M. delirious and restless through the night; pulse 112, soft and full; skin cool and clammy; abdomen slightly tympanitic; violent headache; pain still in the lower part of the abdomen, greatly increased upon pressure; countenance anxious; ordered an injection of castor oil and turpentine, which opened the bowels freely, and relieved both the abdominal pain and pain in the head. Continued the prescription of yesterday and the poultices. 9 P. M. Abdomen still tympanitic, very painful when touched; skin moist and cool; pulse 116, soft and weak; vomiting came on during the day; had taken no diet; drank freely of iced water; ordered 12 doz. leeches to the abdomen; gave lime water, and applied a spice plaster for the vomiting; prescribed ℞ cal. grs. xii., tart. antim. gr. i., P. digitalis grs. ii., P. nit. potass. ℨii., M. ft. chart. No. xxiv., one every two hours. Ordered one of the opium and camphor pills if she should be restless.

17th, 7, A. M. Patient much better; leeches relieved the pain; slept tolerably well; pulse 95; skin soft and pleasant; countenance bright; no pain when left undisturbed; continued the powders of yesterday, and the warm poultices. 11 P. M. Still better; no pain on pressure, but slight soreness; no tympanitis; bowels had been opened three or four times during the day by the powders; vomiting gone; slight nausea; otherwise about as I left her in the morning; continued the powders and directed a couple of the opium and camphor pills through the night, should she be restless.

18th, 8, A. M. Slept well; pulse 90; free from pain; very weak; no sickness when still; calomel touching the gums; discontinued all medicine except an opiate at night in case of restlessness or pain. An opiate was given at night and repeated in two hours.

19th, 8, A. M. Still about as yesterday; had no pain and slept some; no treatment directed, except the poultices and opiate pro re nata; substituted for the cracker panada, crackers and weak tea.

20th. Patient convalescing; continued slowly to improve daily, until the end of the third week, under the same diet, with the addition of buttered toast, when the pulse fell to 76, when she was permitted to sit up in bed and have a somewhat better diet.

This patient was subjected to a rigid antiphlogistic treatment up to the end of the third week; after that time her diet was gradually improved, and at the end of the fourth week she was about her chamber, and in a few days thereafter down stairs to her meals.

The secretion of milk was almost suppressed; the lochia was restored about the 20th; the child was not nursed during fever, but the mother nursed it during convalescence, and continued to do so since her recovery. It is a fine, fat, healthy boy, and I think will do well.

Mrs. H. was a patient in the higher walks of life; surrounded by every comfort and the most careful, watchful nurses. Her temperament is sanguine and nervous; her constitution good, and her habits active, with the exception of the symptoms peculiar to pregnancy, she rarely complains of ill health.

CASE XI. Eliza Gibson, a tall, thin, delicate woman; born in Ireland; æt. 25; married; 2d child; delivered the 16th, M. At the time of confinement sordes on teeth; tongue dry and cracked; pulse 120; respiration 28; seemed comfortable all day; labor easy; lasted two hours.

17th. Slept well all night; complains much of sore throat this morning; upon examination found the fauces and upper portion of the larynx inflamed and œdematous, which was relieved by local applications; pulse 140 and feeble; no abdominal pain or tenderness whatever; gave her a grain of opium, which produced sleep; repeated it at night.

18th. Slept through the night; complains of slight soreness over the uterus. At 10 A. M. felt a little pain; saw her again at 12 M.; pulse 158, tolerably full; respiration 48; took ℥xxiv. of blood, and put on the abdomen 8 doz. leeches; opened her bowels with 10 grains calomel, and a dose of oil; prescribed a grain of calomel and a grain of opium every hour, when awake; slightly tympanitic; ordered poultices to the abdomen.

19th. Pain relieved; tympanitis much increased; continued the treatment; in the evening had an injection of castor oil and turpentine, administered without effect; vomiting commenced; calomel was suspended; larger doses of opium ordered; two and three grains every half hour, until narcotism was produced.

20th. Sinking rapidly; died during the day.

*Autopsy.*—The peritoneum had lost its glossy appearance; no congested condition of the vessels; about half a pint of pure pus in the cavity; right ovary much enlarged; fully the size of an English walnut, and softened; the left one about the size of a partridge egg; uterus but little contracted; walls more than an inch in thickness, and cavity gangrenous.

CASE XII. Margaret Madigan, a short, thick set, red faced woman; born in Ireland; æt. 20; 1st child; delivered on the 13th, after a tedious confinement; 48 hours on the bed; could not impress upon her the importance of keeping quiet; she got up on the second day; was put back to bed; arose again on the third day, and was again put back to bed.

17th. Complained of pain in the abdomen; it commenced swelling; pulse 136; respiration 35; dorsal decubitus; legs drawn up; tongue moist; attempted to bleed her, when she fainted at the loss of ℥iss.; she was a silly, half witted woman, and much frightened; 10 doz. leeches were applied to her abdomen, and a 10 grain dose of calomel with 3 grains of opium given.

18th. Bowels open this morning frequently; so much of a diarrhœa that no more calomel was given; endeavored to arrest the bowels with laudanum injections; opium in large doses—4 grs.—was frequently given; always putting her to sleep.

19th. Abdomen tympanitic; treatment with opium and hot poultices continued until the 21st, when she died.

*Autopsy.*—Abdomen much swollen; cavity of peritoneum contained a quart of pus, its surface dull and pasty, not congested; uterus flaccid, its placental surface in a state of gangrene, and its other portions much inflamed; ovaries slightly enlarged and softened.

Case XIII. Mary Remsler, sthenic; æt. 20; born in Germany; married; 1st child; delivered 13th; seemed comfortable and cheerful up to the 17th. The morning of the 17th complained of acute pain in the iliac regions; had a chill; pulse 132, full and strong; skin pale and hot; was bled 24 oz.; ordered hot poultices to abdomen; gave 10 grains calomel, followed by oil; and prescribed a grain of calomel and a grain of opium every hour; bowels freely opened; bleeding relieved the pain.

18th. Better; pulse 112; less abdominal pain; gave a grain of opium and calomel all day; slept well; the treatment continued for several days with gradual improvement; continued to convalesce rapidly after the 21st, and has been discharged from the house cured; child died of convulsions.

Diet, arrow root.

Case XIV. Martha Nichols, asthenic; born in Germany; æt. 36; single; delivered of her 2d child 14th; was seized with abdominal pains on the 17th; skin pale; hectic flush; pulse 125; very tender belly; ordered 10 dozen leeches to the abdomen and hot fomentations continually; gave 10 grs. of calomel, followed by a dose of castor oil and a grain of calomel, with one of opium every hour.

18th. Abdomen tympanitic, but it subsided after the purging; all pain disappeared after the leeching; treatment continued without change until the 21st, when the application of a large blister to the abdomen removed the remaining tenderness to pressure. The powders were now given every four or five hours; there appeared slight ptyalism, and the patient continued gradually to improve. The pulse sank gradually to its normal frequency by the 28th, and the patient is now convalescent; has not entirely recovered her strength; low diet.

Case XV. Julia Farrell, sthenic; æt. 20; born in Ireland; single; first child; delivered Jan. 28th; easy labor. On the 12th day of confinement lost her child in the ward. The distress produced by the infant's death, and her excited fears in regard to "the fever," induced an attack of hysteria. The paroxysm was violent; it lasted all night and continued until the evening of the next day, (the 10th,) requiring at times several assistants to hold her in bed. These symptoms were treated with the usual remedies.

11th.—Complained of great pain in the abdomen; pulse 100 and weak; would often exclaim "Oh my heart!" When asked to locate the pain of which she most complained, she placed her hand a little below and to the left of her heart; had hectic; a wild vacant stare and anxious expression of countenance; dorsal decubitus; thighs flexed; tongue dry and typhoid. Prescribed 10 grains of calomel and three grains of opium. Bowels had been opened the previous night by an injection. Eight dozen leeches were applied to the abdomen, and she was ordered a grain of opium every hour. At 8, P. M., she had taken seven or eight grains and did not sleep; then prescribed opium in two grain doses; after the first dose she slept a few hours; upon awakening one grain doses were again resorted to, and continued until morning without producing sleep.

11th.—Slight vomiting; pulse very feeble; abdomen very tympanitic and much swollen; pain relieved. Covered the whole abdomen with a blister; gave a dose of castor oil and turpentine; threw it up; vomiting continued occasionally through the day, and was given for that symptom lime water and milk; no sleep; administered an enema of oil and turpentine, which somewhat relieved the tympanitis.

12th.—The opiate was changed to half a grain of morphia in solution, which was given several times during the day. A prescription of calomel and opium, each a grain, was also given every hour. Put a mustard poultice over the blistered abdomen; this somewhat relieved the vomiting.

13th.—Symptoms much the same; patient rather weaker; treatment continued

14th.—Very much prostrated; gave her wine whey and beef essence; powders stopped, sol. morphia two grs. to the ounce substituted; gave a tablepoonful every hour, and repeated the injection of oil and turpentine; obtained some sleep.

15th.—Abdomen not so much swollen; slight pleurisy in right side, relieved by mustard plasters; applied blisters to the iliac region to relieve the recurrent pain and soreness. In this prostrate condition the patient remained until the 20th, manifesting very little change and getting but little sleep. Treatment continued, with an occasional injection of brandy and beef essence.

20th.—Had slept a few hours, better on awakening. Treatment of morphia, wine whey and beef essence continued, with slight improvement each day until the 25th, when she seemed better and had but little vomiting. The patient complaining only of prostration, she was no longer regarded as a puerperal case, and transferred to the medical wards on the 28th. In following up the history of this case, it is proper to say that the vomiting returned after the 28th, when she became much worse; vomiting excessive. Alkalies, acids, aromatics, counter-irritants, blisters dressed with morphia, all were tried, but nothing gave relief. Complains much of weakness.

March 6th.—Injected with a pint and a half of iced water, which relieved vomiting; slept well during the night.

7th.—Was asleep this morning at 10, A. M.; at 8, P. M., much better; has taken whey and beef essence and has not vomited during the day; complains of nothing, and, with the exception of vomiting, has not since the transfer. Has had no lochial discharge since the 10th or 11th day of confinement.

Case XVI. Ellen Campbell, a large, robust woman; born in Ireland; æt. 23; married; 4th child; easy labor; delivered

Jan. 22 ; child weighed ten pounds. This patient did well from the time of her confinement up to the 10th day, when, contrary to orders and the admonitions of assistants, she arose and saw a visitor. The probability is that liquor of some kind had been given her by her *friend*, of which she drank freely. On that night the ward physician was sent for at 12, P. M. ; she could not sleep ; pulse 132 ; respiration not much accelerated, occasionally long and deep drawn ; pain in the head and slight soreness, on pressure, immediately over the uterus. Took ℥xxxv. of blood from arm ; pulse relieved ; headache gone. Gave her a grain of morphia ; slept until morning ; said she felt quite well at 8 next morning. A slight hectic at 1, P. M. ; abdomen swollen and tympanitic ; bowels opened with oil ; countenance much changed, sweating profusely ; lies upon her side ; sinking gradually ; gave morphia one grain every hour ; no sleep. Died that night.

*Autopsy.*—A large amount of pus in the cavity of peritoneum ; ovaries enlarged to the size of a hen's egg, and uterus in a state of gangrene.

This result appears to have been brought about in the short space of 24 hours, and all with scarcely any pain. It must be acknowledged that there was more obscurity about this case than any other contained in this report, and had the result have been favorable, so as to have precluded the proof revealed by the autopsy, the profession at large would scarcely have regarded it as a case of the disease.

Case XVII. Ellen Williams, a robust, healthy woman ; born in Wales ; æt. 22 ; single ; first child ; delivered December 26th ; easy labor.

Jan. 17th.—Three weeks after confinement complained of pain in the lower belly ; fever ; skin hot and dry ; pale ; respiration 51 ; pulse 125, small and corded. Took ℥xxxviii. of blood and applied hot poultices to the belly ; gave 10 grains of calomel followed by salts.

18th.—Better ; pulse 110 ; still pain upon pressure. Prescribed 1 gr. cal., 1 gr. opium every two hours.

19th.—Improving ; pain gradually subsiding, but little tympanitis ; continued treatment ; bowels open.

20th.—Still mending ; continued treatment.

21st.—Convalescing; discontinued medicine. Continued to mend without further bad symptoms until complete recovery. Diet, arrow root.

CASE XVIII. Mary Ramsey, sthenic; born in Scotland; æt. 21; single; first child; delivered Feb. 15; about six hours easy labor; six hours after delivery was seized with cutting pains in the region of the uterus; fever; skin cool and clammy; pulse 132, strong and corded. Venesec. ℥xxxiii. to faintness; pulse reduced and soft; pain relieved; poultices over abdomen; gave 2 grs. opium.

17th.—Slept well until six o'clock this morning; gave ℥j. c. ol., which moved the bowels freely; first time for several days; pulse 110; feels easy; but slight pain upon pressure. Ordered a grain of opium every hour when awake, which was continued for several days with a gradual amelioration of the symptoms, until her recovery. Diet, arrow root.

CASE XIX. Mary Ann Couch, a stout, florid, healthy woman; born in Ireland; æt. 19; married; first child; delivered on Feb. 9th. About 24 hours after delivery complained of sharp cutting abdominal pain; great tenderness on pressure in the iliac regions; slight tympanitis; pale, hot, dry skin; pulse 145, quick and strong; bled her ℥xxviii., which relieved the pain; applied hot fomentations to the abdomen; gave 10 grs. calomel and 2 grs. opium, to be followed in four hours with senna and salts; gave also at night half a grain sul. morphia.

11th.—Rested very well all night; still complained of pain in iliac regions; less fever; pulse 110. Ordered 12 dozen leeches to the abdomen; gave one gr. cal., one gr. opium every hour; tympanitis considerable, for which an enema of oil and turpentine was administered. Continued the powders all day with half a gr. sul. morph. at night.

12th.—Slept well; but little change in symptoms or appearance; pulse 110. Continued treatment. In this condition, with scarcely any change or variation in the pulse, she remained for four days; no pain, but more or less tympanitis; same treatment continued.

16th.—Patient became anemic; complained of great prostration; vomiting commenced; sleeplessness and tympanitis. Gave her quinine and stimuli, also a grain sulph. morphia every hour; blistered the abdomen and dressed it with sulph. morph.; could get no sleep.

These symptoms continued for four days, when the vomiting increased and became black. Had her plunged in a hot mustard bath, in addition to the former treatment. She gradually sank and died on the 20th. An hour or two before death was laboring under violent puerperal mania. Child still living. Low diet in this case until she became anemic.

*Autopsy.*—Found no pus in peritoneal cavity; slight traces of peritonitis; marks of extensive inflammation of the uterus, with gangrene of its cavity; other organs healthy.

CASE XX. Barbara McDermott, asthenic; born in Scotland; æt. 19; single; first child; delivered Feb. 11th; easy labor. This case occurred in the venereal ward, and was the second that appeared in the hospital wing of the institution. This woman had been for some months, and was at the time of delivery, under treatment for primary syphilis. About 36 hours after delivery, complained of pain in the iliac regions and over uterus; pulse 132, and weak; skin pale, cool and clammy; abdomen tympanitic. Applied 10 dozen leeches to abdomen; gave 10 grs. calomel, followed by oil and turpentine; hot fomentations to abdomen constantly. Gave also a grain each of cal. and opium every hour, together with the citras potass. neutral mixture. The cal. and opium with the citras potassæ mixture, were continued for several days; the patient weak but slowly improving. Ten grains of quinine were added to the medicine in the 24 hours. The fourth day of the attack there was slight ptyalism; powders were then given every four hours. Fifth day powders were stopped and a mouth wash ordered.

7th day.—Mouth better and convalescing. Contrary to orders, she got up and walked about the ward; was taken with pain and fever; pulse 125 that night. Abdomen blistered; poultices applied; same powders again administered every hour. Under this treatment the patient improved, and is now up and free from the disease. Her diet was arrow root and rice and milk until last convalescent. Child died in the ward in convulsions.

CASE XXI. Ann Cunningham, a woman of full habit; born in Ireland; æt. 20; single; first child. Labor commenced on the 19th and continued until Thursday the 21st without much progress. Pains came on with great force on the 20th, and hoping, with the natural presentation, it would terminate without artificial assistance, and there being no unpleasant symptoms, she was permitted to remain without interference until Friday night. In view of the violence of the pains, and notwithstanding their frequency, it was determined, after a failure to deliver with the forceps, in consequence of deformity of the pelvis to remove the child by embryotomy. The child being dead, this was done. After delivery the patient slept pretty well from 12, P. M., until morning.

23d. No pain; abdomen much swollen and tympanitic; pulse feeble and very frequent; abdomen blistered and wine administered. Died that night.

*Autopsy.*—Pus in the cavity of peritoneum, about a pint. Cavity of uterus gangrenous.

CASE XXII. Mary Brown; sanguine temperament; born in Ireland; æt. 17; single; 1st child; delivered the 20th; easy labor; delivered at 8, P. M. Complained next morning of sore throat; throat inflamed and œdematous; neck and arms very red; ordered her a blue pill and a gargle to her throat. At 3, P. M. she threw her child out of bed; seemed sullen and out of temper with every one; refused to speak or take medicine. Examined her throat, tonsils much tumefied, a probang dipped in a 30 grain sol. nit. argent. was several times used to it during the afternoon. Evidently insane; pulse very weak; gave wine and carb. ammonia every ½ hour. Died that night, about twenty hours after delivery.

*Autopsy.*—Revealed nothing but the uterus scarcely at all contracted and gangrenous upon its internal surface; child living.

CASE XXIII. Ann Craigan, a stout woman; cook of the ward; born in Ireland; æt. 27; married; 3d child; Jan. 29th, cooked the supper for the patients, washed the dishes; then went into the ward aud delivered herself in 30 minutes of a dead child. This woman appeared to be doing very well, and was anxious to get up on the 3d day; it was with great difficulty she could be kept in bed until the sixth day, when, contrary to orders, she arose; upon hearing the physician's footstep she jumped hurriedly into bed, and was much agitated. That night Dr.

Braxton was called to see her, with a pulse of 112 ; very tender belly ; slightly tympanitic ; and complaining of headache. He prescribed her a grain of opium; the first dose relieved her head, and she slept a few hours. When she awoke her pulse was not so frequent. At 1 P. M. next day, there was much pain and tenderness of abdomen ; pulse 120 stronger than night before. Bled her ℥xx. ; she complained of faintness; stopped the bleeding; pain not removed ; gave her 1 grain opium every hour, until bed-time, when 2 grains were given at once. Slept through the night, waking twice only to ask for water ; each time a powder was given (t. opii gr. i.) Next morning much relieved and better ; opium continued, when awake, and no other treatment resorted to. She gradually improved, and the opium was reduced to 3 grs. a day ; then only at bed-time, and finally, as convalescence appeared, discontinued altogether.

She recovered and left the house about four weeks after confinement.

Case XXIV. Ellen Harnet, a woman of full habit ; born in Ireland ; æt. 20 ; single ; 1st child ; delivered Feb. 9th ; easy labor. The following day was attacked with the usual symptoms of puer. peritonitis; a painful, tender, tympanitic abdomen; pulse 120 to 130 and headache ; bled her about ℥xxxv. when she became faint ; the pulse fell to 100, and she expressed herself relieved entirely of pain, in both the abdomen and head ; gave her 10 grs. calomel and 3 of opium, and had poultices applied to the belly ; slept only partially during the night ; had ½ a grain of morphia 3 or 4 times before morning.

Feb. 11th. Took a dose of oil ; had 10 doz. leeches applied ; abdomen tender ; poultices after the leeching, and gave a grain of calomel and a grain of opium every hour during the day. At night much worse ; very restless ; gave 2 grs. at a dose ; no sleep ; very weak ; gave wine ; continued the stimulus until 9, A. M., the 12th, when she died. No autopsy.

Case XXV. Mary Adams ; sthenic ; born in Ireland ; æt. 24 ; single ; 1st child ; delivered Feb. 25th, at 11 P. M. Twenty-nine hours after delivery found her suffering excruciating abdominal pain ; pulse 136, and strong ; fever ; could not bear the abdomen touched ; dorsal decubitus, with legs drawn up ; took ℥xxx. of blood, and gave 10 grs. calomel with 2 grs. opium, which was thrown up; vomiting excessive; gave two tablespoonfuls liq. morph. sulph. Found her asleep at ½ past 8 next morning, into which she had just fallen; did not awaken her; saw her again at 11, A. M. Still complains of severe abdominal pain; pulse 120, and weak.; great tympanitis; 200 leeches were applied to the abdomen; followed by hot poultices; gave an enema of c. ol. and ol. terebinth.; stomach would retain nothing but sulph. morph.; ordered a grain in solution every hour. 7, P. M. The patient sinking, and died at 2 next morning. Child died 4 days afterwards in convulsions. No autopsy. Sick 36 hours.

Case XXVI. Josephine Smith, a large, muscular woman, in good health; born in Germany; married; æt. 33; 9th pregnancy; delivered Feb. 24th.

27th. Was taken with mental depression, on account of the drunkenness of her husband, and absence from her children, then on their way from Germany.

28th. Bowels moved; some tympanitis; had no purgative; no pain; very little soreness; feared peritoneal mischief; applied 200 leeches. 7, P. M. Pulse 125; no sleep; prescribed a grain of morphia every hour. Slept that night well.

28th. 7, A. M. Awake. A raving maniac; had to be secured to the bed; pulse 110; blistered the abdomen; morphia continued. Slept that night well.

In this state the patient remained until the morning of the 5th of March, when death took place. Throughout the whole course of the disease the skin was pale, but natural; except a day or two before death, when it became cool and clammy; child living. No post mortem.

Case XXVII. Elizabeth Pool; asthenic; born in Pennsylvania; æt. 27; married; 3d child; delivered Feb. 27th. Easy labor; had a slight chill 24 hours after delivery; condition typhoid; pulse feeble, about 100; no prominent symptoms of puerperal peritonitis. Next day pain in abdomen in the region of uterus; 200 leeches applied; was ordered a ½ gr. morphia every hour, which had been regularly administered from the first symptom of chill. Had no purgative; was purged previous to delivery with oil; morphia increased to 1 grain on second day; no sleep. Third day the patient maniacal; fourth day vomiting set in; stopped treatment; died that night at 11 P. M. No post mortem; child died a week afterwards.

I have two other cases to report, but having extended this article to an unwarrantable length, will defer them to some future time. One of them is convalescent; the other I think will be fatal. Making in all 29 cases; out of which were twelve recoveries.

